# Is the minimal invasive anterior rotation spondylodesis a real alternative to corset-treatment?

**DOI:** 10.1186/1748-7161-9-S1-O59

**Published:** 2014-12-04

**Authors:** Stefan Krebs, Tino Riegger, Ioannis Psyllakis

**Affiliations:** 1 Orthopädische Klinik Markgröningen, Markgröningen, Germany

## Introduction

In light cases of scoliosis it is general consensus to apply therapy regimes consisting of physiotherapy and corset wearing. In border cases, between 40 and 50° curvature, opinions vary considerably. The corset supporters often target solely one dimension, specifically the lateral curvature. This is the case where the alternative surgical treatment is possible. In recent times precisely the ventral derotation spondylodesis (VDS) has been developed into a minimal invasive, reduced strain and extremely low risk procedure.

## Materials and methods

Since 2008 we have the results from 71 VDS. In 40 of these there is a following observation period of two years or more.

Measured were not only the pre- and postoperative curvature angle, but also the lateral profiling and balance of the spinal column. Noted were blood loss, surgery duration, complications and stationary duration of stay.

## Results

Preoperative Cobb-angle ø 54° (40-95°), postoperative ø 9° (0-36°). correction loss <3°, blood loss ø 450ml, ø surgery duration 230 minutes.

There was in each case always an improvement of the side profile, particularly as a VDS is to be considered only in case of lordotic deformities. Complications observed was a rod breakage with no pain and no consequences. Otherwise no other complications were observed, especially no neurological ones and no post thoracotomie syndrome, no postoperative disturbance of the lung function.

## Conclusion

The ventral derotation spondylodesis is still a very good treatment method for light single curve scoliosis. Because of the improved surgical but also anesthesiological possibilities over the years, not only the risk of an operation but also the length of the stationary hospital stay are reduced. The costs are considerably below the ones of a lengthy and possibly also unsuccessful conservative treatment with physiotherapy and corset. One should also consider the psychosocial aspects due to stigmatization caused by a long lasting corset therapy. In this regard, a reasonable risk-benefit consideration should be done.

All considered, it appears that even if a residual surgical risk can never be rationalized, in cases of scoliosis in the border area between 40 and 50° precedence should be given to a VDS.

